# Endoplasmic Reticulum Stress Regulates Scleral Remodeling in a Guinea Pig Model of Form-Deprivation Myopia

**DOI:** 10.1155/2020/3264525

**Published:** 2020-06-10

**Authors:** Chengcheng Zhu, Qingzhong Chen, Ying Yuan, Min Li, Bilian Ke

**Affiliations:** ^1^Department of Ophthalmology, Shanghai General Hospital, Shanghai Jiao Tong University School of Medicine, Shanghai, China; ^2^National Clinical Research Center for Eye Diseases, Shanghai, China; ^3^Shanghai Key Laboratory of Ocular Fundus Diseases, Shanghai, China; ^4^Shanghai Engineering Center for Visual Science and Photomedicine, Shanghai, China; ^5^Shanghai Engineering Center for Precise Diagnosis and Treatment of Eye Diseases, Shanghai, China; ^6^Xiamen Eye Centre affiliated to Xiamen University, Fujian, China

## Abstract

**Purpose:**

This study aimed to investigate the role of endoplasmic reticulum (ER) stress in scleral remodeling in a guinea pig model of form-deprivation myopia (FDM).

**Methods:**

Guinea pigs were form deprived to induce myopia. ER ultrastructural changes in the sclera were examined by transmission electron microscopy (TEM). The protein levels of ER stress chaperones, including GRP78, CHOP, and calreticulin (CRT), were analyzed by western blotting at 24 hours, 1 week, and 4 weeks of FD. Scleral fibroblasts from guinea pigs were cultured and exposed to the ER stress inducer tunicamycin (TM) or the ER stress inhibitor 4-phenylbutyric acid (4-PBA). CRT was knocked down by lentivirus-mediated CRT shRNA transfection. The expression levels of GRP78, CHOP, TGF-*β*1, and COL1A1 were analyzed by qRT-PCR or western blotting.

**Results:**

The sclera of FDM eyes exhibited swollen and distended ER at 4 weeks, as well as significantly increased protein expression of GRP78 and CRT at 1 week and 4 weeks, compared to the sclera of the control eyes. *In vitro*, TM induced ER stress in scleral fibroblasts, which was suppressed by 4-PBA. The mRNA expression of TGF-*β*1 and COL1A1 was upregulated after TM stimulation for 24 hours, but downregulated for 48 hours. Additionally, change of TGF-*β*1 and COL1A1 transcription induced by TM was suppressed by CRT knockdown.

**Conclusions:**

ER stress was an important modulator which could influence the expression of the scleral collagen. CRT might be a new target for the intervention of the FDM scleral remodeling process.

## 1. Introduction

The prevalence of myopia continually increases [1–3], and the condition has become a public health problem worldwide. The primary structural abnormality of myopia is the excessive elongation of the ocular globe, which is the result of extracellular matrix (ECM) remodeling of the sclera [4]. The sclera is the outermost layer of the ocular globe and determines the shape and size of the eye. It mainly consists of collagen bundles, in which type I collagen accounts for approximately 95% [5]. Myopic scleral remodeling is a dynamic process that leads to reduced collagen content and thinner collagen fiber bundles, followed by scleral thinning and extension [6, 7]. Although several molecules, such as transforming growth factor-beta (TGF-*β*) [8–10], dopamine [11, 12], retinoic acid [13, 14], and cAMP [15, 16], are known to be involved in scleral changes in myopia, the mechanism underlying myopic scleral remodeling has not been fully elucidated. Clarifying these events is necessary to develop effective therapeutic interventions targeting scleral remodeling and limiting the development of myopia.

Recently, emerging evidence has indicated that endoplasmic reticulum (ER) stress facilitates ECM remodeling in fibrotic diseases [17–19]. ER is a crucial organelle for proper synthesis, maturation, and folding of proteins [20]. A variety of physiological and pathological stimulations or damages may cause perturbation of ER homeostasis, defined as “ER stress.” During ER stress, the ER stress sensors, IRE1, PERK, and ATF6, trigger the unfolded protein response (UPR) [21]. UPR contributes to improved ER protein-folding capacity, restores ER homeostasis, promotes cell survival by expanding the ER size, and upregulates ER chaperones, especially glucose-regulated protein 78 (GRP78) [22, 23]. If the adaptive responses of ER are inadequate to restore ER homeostasis, sustained UPR signaling induces the expression of UPR-associated proapoptotic transcriptional regulators, such as C/EBP homologous protein (CHOP), leading to cell apoptosis and death [24, 25]. Whether ER stress participates in myopia-related scleral remodeling has not yet been defined.

Calreticulin (CRT) is a Ca^2+^-binding chaperone localized in the ER lumen, playing a crucial role in protein folding, calcium homeostasis, and many other biological processes [26, 27]. CRT has been shown to regulate collagen transcription, trafficking and processing in embryonic fibroblasts [28]. Moreover, CRT was also found to be an important ER stress chaperone, which was required for ER stress and TGF-*β*1-induced collagen production in many fibrotic diseases [29–31]. As CRT is a key mediator of ECM production, therapeutic approaches targeting CRT have been explored in promoting wound healing [32]. However, it is still unclear whether ER stress response is regulated by CRT in FDM scleral remodeling.

In this study, in order to clarify the role of ER stress and CRT in FDM, ER stress response was investigated *in vivo* and *in vitro* in a guinea pig model of the disease.

## 2. Materials and Methods

### 2.1. Form-Deprivation Myopia Model

All procedures conformed to the Association for Research in Vision and Ophthalmology (ARVO) Statement for the Use of Animals in Ophthalmic and Vision Research. The experimental protocols were approved by the ethics committee of the Shanghai General Hospital, Shanghai Jiao Tong University School of Medicine. Three-week-old pigmented guinea pigs (*n* = 20) were bred at the Laboratory Animal Center under a 12-h light-dark cycle. They were randomly divided into three groups, which was given 24 hours, 1 week, and 4 weeks of FD, respectively. One eye was randomly selected from each guinea pig and covered with a translucent diffuser to induce form-deprivation myopia (FDM), as previously described [33], while the other eye served as the untreated control.

Refraction and axial length were determined before and after treatment. The refractive errors were examined using an automated infrared photorefractor, as previously described [33]. The axial lengths were measured by A-ultrasonic scanning (KN-1800, KangNing, China). All examinations were performed by two doctors independently and repeated three times to obtain the average value.

### 2.2. Transmission Electron Microscopy (TEM)

Both the control and FD eyes after 4 weeks of FD (n=5) were enucleated and fixed in 3% glutaraldehyde, incubated at 4°C for 24–48 hours and placed in 1% osmium. After dehydration, the tissues were embedded in an epoxy resin mixture at 60°C for 48 hours. Sections (100 nm) were taken from the posterior sclera and placed on copper mesh grids for TEM examination (FEI Tecnai G2 Spirit, USA).

### 2.3. Primary Culture of Scleral Fibroblasts

Primary cultures of scleral fibroblasts were obtained from whole sclera explants of 2-week-old guinea pigs. The scleral tissue was cut into tissue blocks of 1 × 1 × 1 mm^3^ under sterile conditions and carefully placed into separate flasks in Dulbecco's modified Eagle medium (DMEM; High Glucose, Gibco, USA) containing 10% fetal bovine serum (FBS; Gibco, USA) and 1% penicillin/streptomycin (Gibco, USA). Fibroblast cultures were incubated at 37°C in a humidified atmosphere containing 5% CO_2_ until confluent. The medium was replenished twice a week. Cells at 80% conﬂuence were passaged by using 0.25% trypsin-EDTA (Gibco, USA). The cells were identified by vimentin detection, as previously described [33]. Fibroblasts between 3 and 5 passages were used for the experiments. Cells were washed and cultured with DMEM (High Glucose) without FBS for 24 hours and then incubated with basal medium plus 0.1 *μ*M tunicamycin (TM; Cell signaling technology, USA), 2.5 mM 4-phenylbutyric acid (4-PBA; Sigma-Aldrich, USA), and with 0.1 *μ*M TM + 2.5 mM 4-PBA to extract proteins and total RNA, respectively.

### 2.4. Construction and Transfection of the Lentiviral shRNA Vector

The shRNA and lentivirus were constructed by Sangon Biotech (Shanghai, China). The small interfering RNA (siRNA), 5′-GGGTCGAATCCAAACACAAGT-3′, targeting the guinea pig calreticulin (CRT) gene was selected for constructing the lentiviral shRNA vectors. The target shRNA sequences were synthesized and cloned into a lentiviral vector, and the sequence 5′-TTCTCCGAACGTGTCACGT-3′, with no significant homology to any guinea pig gene, was cloned into the same vector and used as the negative control. For cell transfection, primary cultures of scleral fibroblasts were seeded at a density of 10 × 10^5^ in 6-well plates and transduced with the lentiviral particles.

### 2.5. Quantitative Real-Time Reverse Transcription-Polymerase Chain Reaction (qRT-PCR)

Total RNA of cells was harvested with the Trizol reagent and isolated as described by the manufacturer's specifications. Reverse transcription was performed using the PrimeScript RT reagent kit (Takara Bio, Japan). SYBR Premix Ex Taq™ II (Takara Bio, Japan) was used to perform real-time PCR, and an Applied Biosystems ViiA™ 7 system (Life technologies, USA) was used for detection. Primers for guinea pig GRP78 (forward: CTCCGTTCAGCAAGACATCA, reverse: AGCCTCAGCAGTTTCCTTCA, 160 bp), CHOP (forward: CCTTTCTCCTTCGGGACACT, reverse: CTCTTCATTTCCAGGGGGTAA, 120 bp), CRT (forward: CGGTGAAGCATGAGCAGAACATTG, reverse: CGAGTCTCCGTGCATGTCCTTC), TGF-*β*1 (forward: CCCAGAGTGGTTGTCCTTTG, reverse: CGGAGCGTGTTATCTTTGCT, 123 bp), COL1A1 (forward: TGGGTCCTACTGGCAAACAT, reverse: TCACCAACCTCTCCCTTGTC, 133 bp), and GAPDH (forward: TCAAGAAGGTGGTGAAGCAG, reverse: CGTCAAAAGTGGAAGAATGG, 117 bp) were synthesized by Sangon Biotech (Shanghai, China). Relative mRNA expression levels were calculated by using the 2^−ΔΔCT^ method from the Ct values of the respective mRNAs relative to that of GAPDH [33].

### 2.6. Western Blotting

The sclera tissues (*n* = 5, each group) or cultured fibroblasts were sonicated in the RIPA lysis buffer (Beyotime Biotechnology, Jiangsu, China) containing a protease inhibitor. After centrifugation, the supernatants were collected. Proteins at equal concentration were separated by 10% SDS-PAGE and transferred to PVDF transfer membranes (Millipore Corporation, Temecula, CA). The membranes were incubated in 5% milk/TBST (20 mM Tris-HCl, pH 7.6, 137 mM NaCl, 0.1% Tween 20), followed by overnight incubation at 4°C with the appropriate dilutions of GRP78 (Abcam, UK), CHOP (Cell signaling technology, USA), CRT (Cell signaling technology, USA), and GAPDH (Santa Cruz, USA) primary antibodies. After a rinse in TBST, the membranes were incubated for 1 h with the horseradish peroxidase-conjugated secondary antibody against rabbit or mouse IgG (Jackson ImmunoResearch, USA). The proteins were visualized by enhanced chemiluminescence (Pierce, USA). The density of the bands was analyzed by a Gel-Pro Analyzer.

### 2.7. Statistical Analysis

Data were expressed as the mean ± standard deviation (SD). Student's paired *t*-test was used to analyze the differences between right and left eyes. One-way ANOVA was used to compare the cells before and after the intervention. *P* < 0.05 was considered statistically significant.

## 3. Results

### 3.1. Refraction and Axial Length Measurements of Guinea Pigs

**Figure 1:**
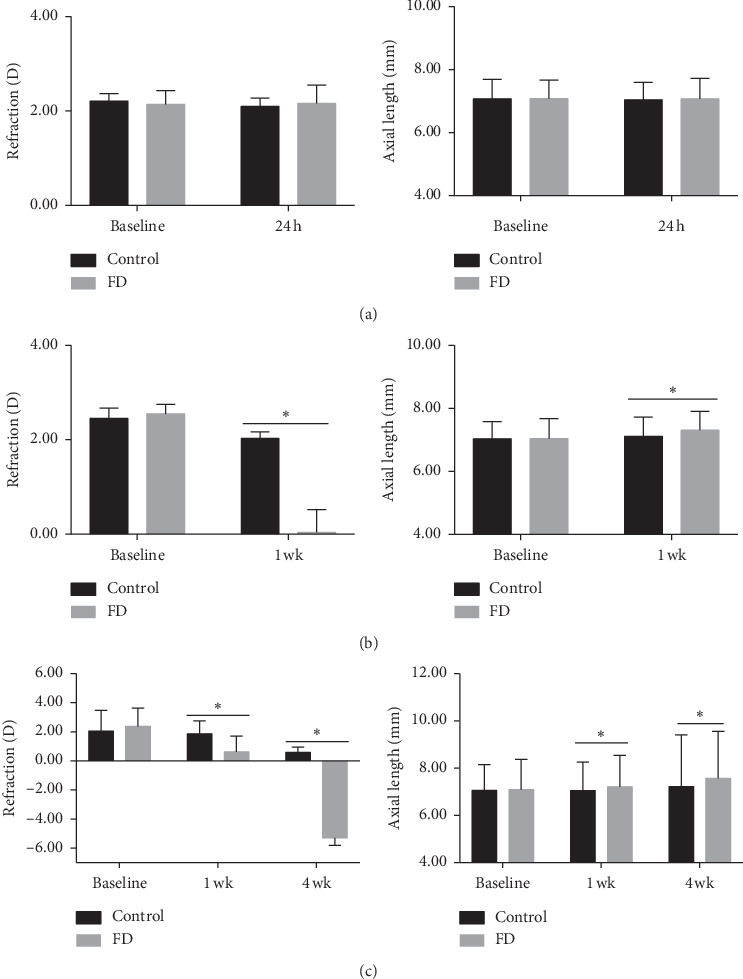
Refraction and axial length in control and FD eyes in 24-hour (a), 1-week (b), and 4-week (c) treatment groups. Data are expressed as the mean ± SD. ^*∗*^*P* < 0.05.

There were no significant differences in refraction or axial length between control and FD eyes of guinea pigs at the beginning of the experiment (*P* > 0.05). After 1 week and 4 weeks of FD, significant differences were induced in refraction and axial length between the control and FD eyes (*P* < 0.05, Figure 1).

### 3.2. ER Stress Was Activated in the Sclera of FDM Eyes

**Figure 2:**
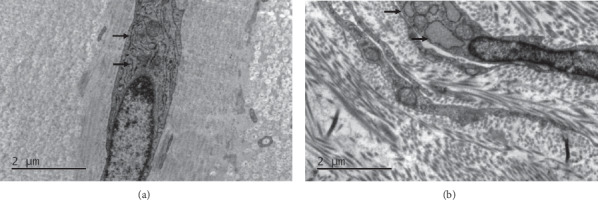
Morphological changes of ER detected by transmission electron microscopy in the sclera of control and FD eyes. (a) Control; (b) FDM. The black arrows indicate the ER.

**Figure 3:**
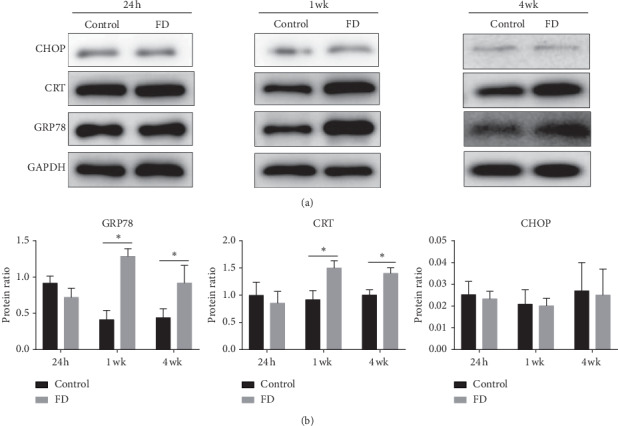
The protein expression of GRP78, CHOP, and CRT in the sclera of the control and FD eyes. Data are expressed as the mean ± SD. ^*∗*^*P* < 0.05.

To assess whether ER stress was activated in the sclera of FDM, we investigated ER morphologic changes by TEM and examined the expression of ER stress-associated proteins by western blotting in control and FD eyes. After 4 weeks of treatment, a swollen and distended ER was observed in the sclera of FD eyes, while it showed normal ER morphology in control eyes (Figure 2). Moreover, protein levels of GRP78 and CRT had no significant differences between control and FD eyes after 24 hours of FD, while they were significantly increased in the FD eyes compared to the control eyes after 1 week and 4 weeks of FD (*P* < 0.05, Figure 3). No significant difference in CHOP expression was detected between control and FD eyes after 24 hours, 1 week, and 4 weeks of FD (*P* > 0.05, Figure 3).

### 3.3. TM Induced ER Stress in Guinea Pig Scleral Fibroblasts

**Figure 4:**
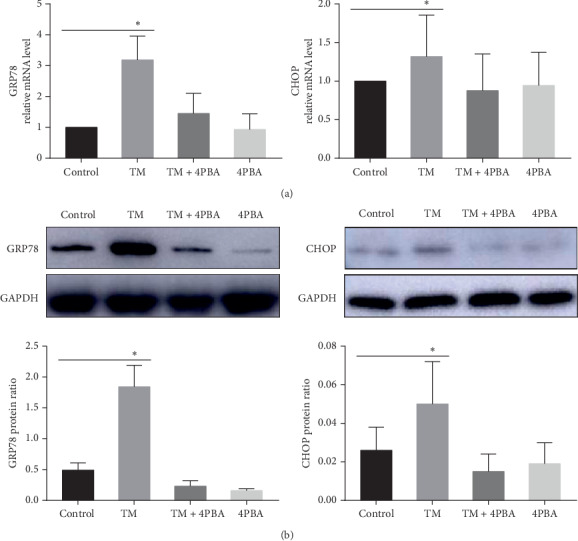
The mRNA (a) and protein (b) expression of GRP78 and CHOP in scleral fibroblasts treated in the absence or presence of TM, TM + 4-PBA, and 4-PBA. Data are expressed as the mean ± SD. ^*∗*^*P* < 0.05.

To explore the role of ER stress in the sclera, we cultured primary guinea pig scleral fibroblasts and treated them with a chemical inducer (TM) or an inhibitor (4-PBA) of ER stress. As shown in Figure 4, TM induced mRNA and protein expression of GRP78 and CHOP at 24 hours. The ER stress induced by TM was suppressed by 4-PBA (Figure 4).

### 3.4. ER Stress Regulated Transcription of COL1A1 and TGF-*β*1 in Guinea Pig Scleral Fibroblasts

**Figure 5:**
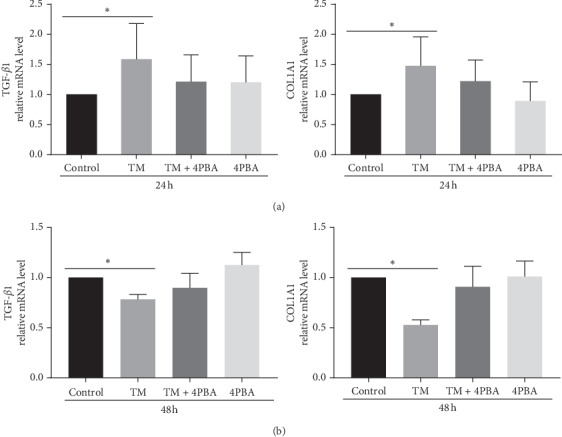
The mRNA expression of TGF-*β*1 and COL1A1 in scleral fibroblasts after treatment with TM, TM + 4-PBA, and 4-PBA for 24 hours (a) and 48 hours (b). Data are expressed as the mean ± SD. ^*∗*^*P* < 0.05.

To further investigate the impact of ER stress on the collagen, we evaluated the mRNA expression levels of COL1A1 and TGF-*β*1 after TM treatment. Treatment with TM upregulated TGF-*β*1 and COL1A1 mRNA expression at 24 hours but downregulated TGF-*β*1 and COL1A1 mRNA expression at 48 hours. 4-PBA inhibited the change of TGF-*β*1 and COL1A1 transcription after TM stimulation (Figure 5).

### 3.5. CRT Mediated Transcription of COL1A1 and TGF-*β*1 during ER Stress in Scleral Fibroblasts

**Figure 6:**
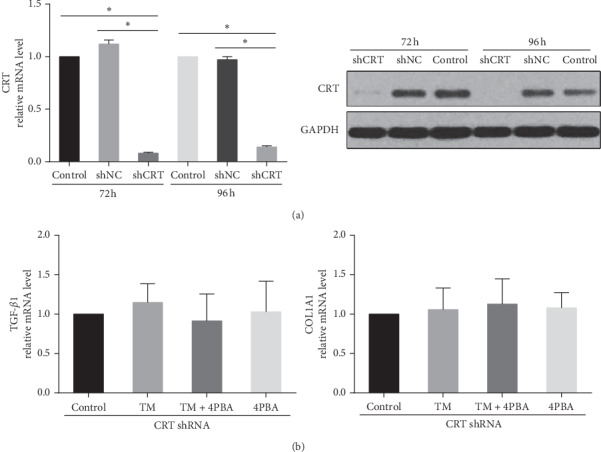
(a) CRT mRNA and protein expression in scleral fibroblasts transfected with lentivirus CRT shRNA. NC: negative control. (b) The mRNA expression of TGF-β1 and COL1A1 in CRT knockdown scleral fibroblasts after treatment with TM, TM + 4-PBA, and 4-PBA for 24 hours. Data are expressed as the mean ± SD. ^*∗*^*P* < 0.05.

We evaluated the change of COL1A1 and TGF-*β*1 transcription in CRT knockdown scleral fibroblasts after TM stimulation. The mRNA and protein expression of CRT were significantly inhibited in the CRT shRNA group compared to both the control and the negative control shRNA group, after 72 h and 96 h of lentivirus transfection (*P* < 0.05, Figure 6(a)). In CRT knockdown scleral fibroblasts, TM stimulation did not upregulate the mRNA expression level of TGF-*β*1 and COL1A1 ( *P*＞0.05, Figure 6(b)).

## 4. Discussion

The development of myopia is closely associated with scleral remodeling, but the factors that regulate this process are not fully established. In the present work, we identified that ER stress was triggered during scleral remodeling in FDM. We also demonstrated in scleral fibroblasts that ER stress induced early transcription of COL1A1 and TGF-*β*1, while sustained ER stress inhibited their levels. CRT knockdown suppressed the transcriptional change of COL1A1 and TGF-β1 by TM stimulation. Such changes suggest ER stress to be an important modulator of scleral ECM production. To the best of our knowledge, this is the first study to elucidate such a role for ER stress during myopia.

The *in vivo* experiments showed that GRP78 was significantly increased in the sclera of FD eyes both at 1 week and 4 weeks, while CHOP showed no significant difference. Previous studies have reported that GRP78 is an ER chaperone protein and a regulator of UPR, promoting protein folding [34]. The induction of GRP78 is an established general indicator of ER stress [35]. Moreover, abnormal morphological changes, including swollen and dilated ER, were observed in the sclera after 4 weeks of FD, also suggesting that ER stress was induced. Taken together, these results demonstrated that ER stress was involved in the scleral remodeling of form-deprivation myopia. CRT, a mediator of ER stress-induced collagen production [30], was also increased in the sclera of FDM eyes. This suggested that FDM-induced ER stress might affect the production of extracellular matrix.

ER stress has been reported to decrease collagen production in chondrocytes and dermal fibroblasts [36, 37] while promote collagen production in lung fibroblasts [38–40], hepatic stellate cells [41], and myocardial cells [42]. In this study, we found that TM stimulation increased COL1A1 and TGF-*β*1 levels at 24 hours but reduced their expression at 48 hours in scleral fibroblasts. It has been well established that the levels of TGF-*β* and collagen were reduced during FD [10, 43]. However, we found that, *in vitro*, chemically-induced ER stress had a time-dependent bidirectional regulation effect on collagen transcription. A similar ER stress-mediated bidirectional regulation of NF-*κ*B was reported previously [44]. The reason may be that ER stress firstly leads to cellular translational and transcriptional changes to promote cell survival, but prolonged ER stress results in cellular dysfunction and apoptosis by activation of different UPR signaling branches, which is consistent with the previous theory [22, 45]. According to time-varying responses of ER stress induced by TM *in vitro*, we speculated that *in vivo* FD induced pathological conditions that decreased the scleral collagen, while ER stress was triggered to serve a potential compensatory role in scleral collagen reduction. But prolonged ER stress ultimately contributed to decrease of collagen and myopia development. However, this effect of ER stress on onset and progression of FDM need to be further verified in animal models by genetic predisposition or preconditioning intervention [46]. In general, we demonstrated the characteristic of ER stress response *in vitro*, which established the foundation for the further study.

Moreover, we also found CRT was a potential mediator between ER stress and collagen expression. CRT is a Ca^2+^-binding ER chaperone that ensures proper protein folding. It is induced by ER stress and plays critical roles at collagen production, including expression, secretion, processing, and deposition in fibrotic diseases [28, 29, 47]. Zimmerman reported that CRT was required for TGF-*β* and ER stress-stimulated collagen production in mouse embryonic fibroblasts previously [29]. However, the role of CRT had not been reported in myopic scleral remodeling. In this study, we found that after CRT was knocked down by lentivirus-mediated CRT shRNA, TM-induced transcription of COL1A1 and TGF-*β*1 was inhibited in scleral fibroblasts. It demonstrated that CRT was a critical mediator between ER stress and collagen *in vitro*. In vivo, CRT was upregulated in the sclera after 1 week and 4 weeks of FD, but it showed a descending trend at 4 weeks of FD compared with 1 week. These results implied that the upregulation of CRT during rapid change of ER stress may be a compensation for collagen decrease in the FDM sclera, which need further investigation.

There are some limitations in this study. Firstly, we verified that CRT was a potential mediator between ER stress and collagen expression *in vitro*. However, its role during FDM scleral remodeling *in vivo* is still not clarified, which is our further research orientation. Secondly, ER stress induces complex molecular events though different UPR signaling pathways. They may have opposing effects on scleral remodeling and regulate the process of FDM. So, it is necessary to study the role of other chaperones in ER stress.

In summary, our findings demonstrated that ER stress was activated in the sclera of myopia models. ER stress was found to regulate collagen production through CRT in scleral fibroblasts. Our findings provided new insights into the mechanisms of scleral remodeling in myopia and suggested that CRT may be a potential target for treatment of myopia.

## Data Availability

The data used to support the findings of this study are available from the corresponding author upon request.
